# Multiomics Analysis Reveals Novel Genetic Determinants for Lens Differentiation, Structure, and Transparency

**DOI:** 10.3390/biom13040693

**Published:** 2023-04-19

**Authors:** Joshua Disatham, Lisa Brennan, Ales Cvekl, Marc Kantorow

**Affiliations:** 1 Charles E. Schmidt College of Medicine, Florida Atlantic University, Boca Raton, FL 33431, USA; jdisatha@health.fau.edu (J.D.); lbrenna6@health.fau.edu (L.B.); 2 Departments of Ophthalmology and Visual Sciences and Genetics, Albert Einstein College of Medicine, Bronx, NY 10461, USA; ales.cvekl@einsteinmed.edu

**Keywords:** ATAC-seq, CUT&RUN, RNA-seq, multiomics, bisulfate sequencing, chromatin, gene regulation, development, differentiation

## Abstract

Recent advances in next-generation sequencing and data analysis have provided new gateways for identification of novel genome-wide genetic determinants governing tissue development and disease. These advances have revolutionized our understanding of cellular differentiation, homeostasis, and specialized function in multiple tissues. Bioinformatic and functional analysis of these genetic determinants and the pathways they regulate have provided a novel basis for the design of functional experiments to answer a wide range of long-sought biological questions. A well-characterized model for the application of these emerging technologies is the development and differentiation of the ocular lens and how individual pathways regulate lens morphogenesis, gene expression, transparency, and refraction. Recent applications of next-generation sequencing analysis on well-characterized chicken and mouse lens differentiation models using a variety of omics techniques including RNA-seq, ATAC-seq, whole-genome bisulfite sequencing (WGBS), chip-seq, and CUT&RUN have revealed a wide range of essential biological pathways and chromatin features governing lens structure and function. Multiomics integration of these data has established new gene functions and cellular processes essential for lens formation, homeostasis, and transparency including the identification of novel transcription control pathways, autophagy remodeling pathways, and signal transduction pathways, among others. This review summarizes recent omics technologies applied to the lens, methods for integrating multiomics data, and how these recent technologies have advanced our understanding ocular biology and function. The approach and analysis are relevant to identifying the features and functional requirements of more complex tissues and disease states.

## 1. Introduction: The Eye Lens as a Model System for Unbiased Multiomics Analysis of Gene Regulatory Mechanisms

**Figure 1:**
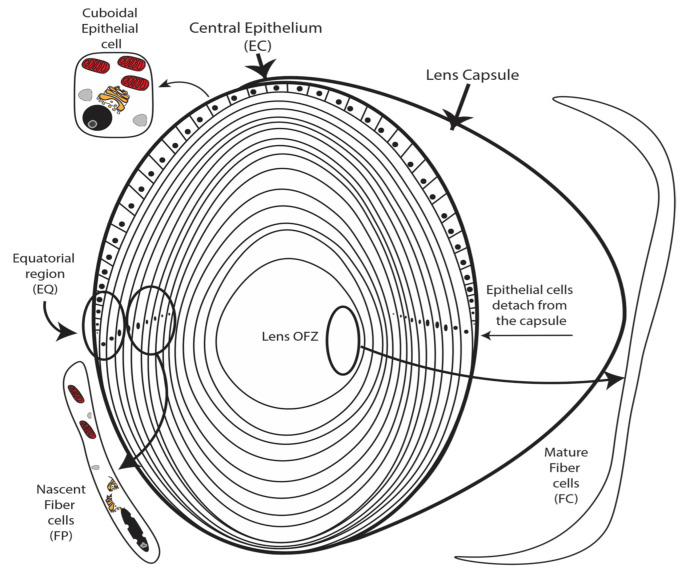
The lens is composed of a layer of undifferentiated epithelial cells that overlie a core of elongated fully differentiated organelle-free transparent fiber cells. The process of lens differentiation is characterized by the dissociation of epithelial cells from the lens capsule (indicated), elimination of all cellular organelles (indicated), and elongation to the form the core of fully differentiated fiber cells forming the core lens organelle-free zone (OFZ) (indicated). Degradation of organelles is a hallmark feature of lens cell differentiation (indicated). Lens fiber cell nuclei condense before degradation (indicated).

A long-studied model for identifying mechanisms controlling organ development and cellular differentiation is the ocular lens that functions to focus light on the retina. Key to its utility for revealing novel mechanisms of development and differentiation, the lens is composed of two cell types including a single layer of undifferentiated cuboidal epithelial cells that overlay a core of elongated terminally differentiated fiber cells (Figure 1). During embryogenesis and throughout life, lens epithelial cells continuously differentiate into lens fiber cells through a tightly regulated differentiation program [1,2,3,4]. Lens cell differentiation is initiated in the germinal zone of the lens (Figure 1) where quiescent epithelial cells irreversibly withdrawal from the cell cycle [3,5]. Following cell cycle exit, nascent fiber cells then undergo a series of terminal remodeling and maturation steps including cellular elongation (Figure 1), expression of highly abundant fiber cell proteins, and the hallmark elimination of cellular organelles to achieve mature lens structure and transparency. These fully mature fiber cells constitute the core of the lens in a region of the lens called the organelle-free zone (OFZ) (Figure 1). Failure of lens epithelial cells to differentiate into fiber cells results in major lens structural defects and loss of transparency [6].

Numerous elegant studies conducted over many years have identified important growth factors, signaling pathway, and downstream transcriptional mechanisms within the lens fiber cell nuclei regulating lens differentiation [1,7,8,9]. These pathways include but are not limited to Wnt [10,11,12,13,14,15], Notch [16,17,18,19,20,21], BMP [22,23,24,25,26,27,28,29,30], FGF-MAPK [31,32,33,34,35,36,37,38,39,40,41,42], and Hippo pathways [43,44]. Key transcription factors include Pax6 [45,46,47,48,49,50], Prox1 [49,51,52,53,54], Hsf4 [55,56,57], Gata3 [58,59,60], FoxE3 [61,62,63,64,65,66,67], c-Maf [48,68,69,70,71], MafG and MafK [72,73], N-Myc [74,75], Pitx3 [76,77,78,79,80,81], and HIF1α [82,83,84,85]. Despite these important findings, the entirety of mechanisms regulating lens differentiation have not been entirely elucidated. A limitation to identifying the range and spectrum of lens differentiation pathways and requirements has been an inability to examine lens differentiation from a genome-wide perspective.

Insights into the genome-wide gene expression occurring during lens development and differentiation have been generated using mainly chicken and mouse models. Other detailed studies of frog, zebrafish, and medaka fish models have also been reported [86,87,88]. Different lens models offer unique advantages since different species lenses evolved for different environments and diurnal/nocturnal conditions. Since the majority of multiomics studies to date have focused on the chicken and mouse lens, these species will be highlighted in this review.

The main difference between the chicken and mouse lens is the higher accommodative power of chicken and avian lenses that are adapted to providing the acute vision needed for flight and survival. By contrast, nocturnal mice have much more rigid lenses. The main lens structural proteins responsible for its transparency and light refraction are crystallins [89,90,91,92]. While members of the α- and β/γ-crystallin subfamilies are present in all vertebrate genomes, lens evolution in some species has produced taxon-specific crystallins via “gene sharing” [93]. In chicken/avian lenses, this is illustrated by use of argininosuccinate lyase gene as the most abundant pair of δ1- and δ2-crystallin genes [94]. A lens-specific enhancer evolved in their intron 3 [95], and studies have demonstrated its direct regulation via Pax6, Sox2, and MafA (L-Maf) bindings [96,97,98,99,100]. Studies of chicken/mouse c-Maf with this enhancer remain to be conducted. At the genome level, chickens lack the cluster of six γ-crystallin genes (Cryga, Crygb Crygc, Crygd, Cryge, and Crygf) located on mouse chromosome 1. These crystallins are abundant in the nucleus (or OFZ) of the mouse lens that make it very rigid. The human syntenic region of chromosome 2 contains three pseudogenes, ΨγG, ΨγE and ΨγF. Thus, human lenses are softer due to reduced expression of γ-crystallins. Both human and avian lenses need to accommodate large distances relative to a mouse lens. The “core” gene regulatory network (GRN) of mouse crystallin gene expression is a feed-forward loop in which Pax6 directly controls the expression of c-Maf and both Pax6 and c-Maf directly bind to promoters/enhancers of the α- and β/γ-crystallin genes [48]. Comparative studies of individual crystallin promoters in different species are thus important for our understanding of how gene expression affects the evolutionary properties of the lenses of various species. 

Recent advances in multiomics approaches including RNA-seq, assay for transposase accessible chromatin with sequencing (ATAC-seq), whole-genome bisulfite sequencing (WGBS), chromatin immunoprecipitation with sequencing (ChIP-seq), and cleavage under targets and release using nuclease (CUT&RUN) have enabled the assembly of genome-wide data encompassing a wide-range of features and regulatory pathways important for lens differentiation [85,101,102,103,104]. Analysis of these data has allowed for the integration of differentiation state-specific transcriptional networks with essential gene expression-specific chromatin features that have confirmed the importance of a multitude of previously identified lens differentiation pathways and have identified entirely novel pathways as described below. This integrated approach is applicable to identifying differentiation requirements of more complex organs and tissues. In this review, we summarize the high-throughput genomic techniques, model systems, and analytical methods employed to unravel the complexity of lens differentiation with an emphasis on how this technology can be broadly applied.

## 2. Bulk Transcriptome Analysis of the Lens via RNA-seq

**Figure 2:**
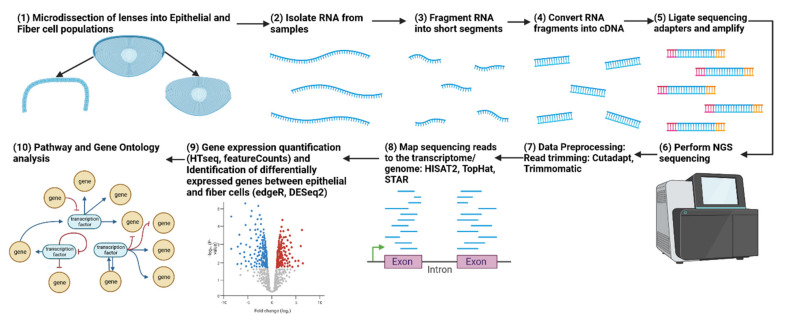
Workflow for an RNA-seq experiment comparing lens epithelial cells and fiber cells. The figure illustrates the sequential steps involved in the RNA-seq experiment, beginning with the isolation of lens epithelial and fiber cells, followed by RNA extraction, library preparation, sequencing, data preprocessing, differential gene expression analysis, and downstream functional enrichment analyses to identify the molecular mechanisms controlling lens differentiation. *Adapted from “RNA Sequencing” by BioRender.com (2023)*. Retrieved from https://app.biorender.com/biorender-templates.

RNA-seq is a powerful tool for analyzing gene expression during cellular differentiation due to its ability to provide the high-throughput, unbiased, and quantitative measurement of transcriptome-wide gene expression. The RNA-seq process involves isolation of total RNA from the sample of interest, cDNA synthesis, and sequencing of the generated cDNA using high-throughput sequencing technology (Figure 2). RNA-seq technology emerged in the late 2000s as an alternative to microarrays and EST (expressed sequence tags) sequencing. The first RNA-seq method of mammalian transcriptomes was developed by Mortazavi et al. in 2008, which involved deep sequencing of short cDNA fragments generated from RNA samples using next-generation sequencing (NGS) platforms [105]. The sequencing data obtained from RNA-seq experiments can be analyzed to measure gene expression levels, identify differentially expressed genes, detect alternative splicing events, and quantify transcript isoforms. The data can be aligned to a reference genome or de novo assembled into a transcriptome. The expression levels of genes can be measured using different methods, such as reads per kilobase per million mapped reads (RPKM), fragments per kilobase of transcript per million mapped reads (FPKM), or transcripts per million (TPM). The ability of RNA-seq to measure gene expression levels in a wide range of tissues and under different conditions has made it an indispensable tool for studying gene regulation, developmental biology, and disease mechanisms [106,107,108,109,110].

### 2.1. Analysis of Lens Epithelium and Fiber Cell Compartments in Chicken and Mouse Lenses by RNA-seq

In the lens, RNA-seq has been used to examine changes in gene expression during lens development and differentiation. Studies that have used RNA-seq analysis of lens differentiation mechanisms can be broadly split into two categories. RNA-seq studies comparing epithelial cells versus fiber cell transcriptomes provide insight into the gene expression changes that occur during the differentiation process [102,111,112,113]. These studies can reveal genes, transcription factors, and molecular pathways that underlie lens differentiation during the transition from epithelial cells to fiber cells as well as those mechanisms critical for maintaining lens structure and transparency. By contrast, RNA-seq studies comparing wild-type versus mutant lens transcriptomes aim to identify the molecular changes that occur due to genetic mutations that affect lens differentiation [53,60,75,114,115,116,117,118,119]. By identifying the genes and pathways that are dysregulated in these mutant lenses, researchers can gain a better understanding of the molecular mechanisms underlying lens development and the genetic factors that contribute to cataract formation. The information from the two types of RNA-seq studies can be used to generate hypotheses about the molecular mechanisms underlying lens differentiation which can then be tested using functional assays or other experimental approaches.

Several RNA-seq studies in both chicken and mouse model systems have been conducted in the past decade (Table 1). A study by Chauss et al. in 2014 aimed to identify the entire transcriptional complement of genes expressed in specific regions of differentiation of the embryonic day 13 (E13) *Gallus gallus* (chicken) eye lens. These differentiation-state-specific regions contain morphologically distinct cell populations including a monolayer of undifferentiated epithelial cells at the center of the lens anterior (EC cells), epithelial cells at the lens equator that withdraw from the cell cycle to initiate differentiation (EQ cells), a zone of newly formed fiber cells at the lens cortex that undergo a series of remodeling events including elongation (FP cells), and finally, a core of elongated fiber cells from which all organelles are eliminated and which make up the bulk of the lens (FC cells) [2,3,9,84,111,120,121,122,123,124,125,126,127,128,129]. Similarly, a subsequent study by Disatham et al., 2022 conducted a multiomics analysis of E13 chicken lenses microdissected into lens epithelial and fiber cells to compare transcriptomic differences, chromatin accessibility differences, and DNA methylation differences specific for lens differentiation [102]. A component of this study utilized RNA-seq to directly compare gene expression patterns between lens epithelial cells and lens fiber cells. A study by Hoang et al., 2014 was the first to use RNA-seq on a newborn mouse lens microdissected into lens epithelial and fiber cells to identify specific patterns of gene expression [113]. Finally, an elegant 2018 study by Zhao et al. involved a comprehensive spatiotemporal analysis of lens gene expression patterns between epithelial and fiber cells at multiple mouse developmental stages, including E14.5, E16.5, E18.5, and P0.5 [112]. 

Multiple notable genes exhibit preferred expression in lens epithelial cells compared to lens fiber cells across both chicken and mouse model systems (Table 1). The genes in these studies have a wide range of established functions critical for lens structure, homeostasis, and transparency. Some notable epithelial cell preferred genes that have been highlighted in both mouse and chicken studies include three components of Notch signaling (Notch2 receptor and transcription factors Rbpj and Hes1) [16,17,18] and transcription factor Pax6 [49,98,130]. Consistently, some notable fiber cell preferred genes conserved in both mice and chicken include beaded filament proteins (BFSP1, BFSP2) [126], mitophagy genes (BNIP3) [121,131], macroautophagy genes (MTOR) [132], and transcription factors (HSF4, and PROX1) [51,53,55,56,57]. An integrated statistical comparison of the most recent studies in chicken [102] and mice [112] is provided in Section 5 of this review to identify the degree of overlap between these model systems and demonstrate how these data could be used to identify established and novel functional mechanisms required for lens differentiation conserved across species.

Collectively, these studies identify evolutionarily conserved gene expression patterns between lens epithelial and fiber cells, suggesting their functional importance to the lens differentiation mechanism. By identifying conserved genes that are differentially expressed during the transition from proliferating epithelial cells to differentiated fiber cells, researchers can better understand the molecular mechanisms and regulatory pathways underlying this process. Some notable conserved pathways and ontologies highlighted by these studies are discussed in the next section. Overall, RNA-seq has allowed for a more comprehensive and accurate analysis of the lens transcriptome and has provided insights into the molecular mechanisms underlying lens differentiation.

### 2.2. Signaling Pathways Inferred from RNA-seq Data

One advantage of comparing lens differentiation mechanisms identified in mice and chicken model systems is the ability for researchers to distinguish between genes and pathways that are functionally critical for lens differentiation versus the genes and pathways that appear to be species-specific. Comparative studies of gene expression in mouse and chicken lens epithelial and fiber cells have identified numerous conserved signaling pathways involved in lens differentiation (Table 2). The studies in mice lenses [112] and chicken lenses [103] have identified the enriched signaling pathways and ontologies associated with lens epithelial cells compared to those associated with lens fiber cells. Some of the notable signaling pathways associated with epithelial cell genes include cell cycle, extracellular matrix organization, cell adhesion, epithelial–mesenchymal transition, and Notch signaling. In contrast, the pathways associated with fiber cell genes include oxidative phosphorylation, mTOR signaling, lipid metabolism, apoptosis, protein ubiquitination, autophagy, and intermediate filament organization. Additionally, mechanisms related to the regulation of mitochondrial populations in lens fiber cells have been identified in both mouse [112] and chicken lenses [111].

It should be noted that the mouse studies by Zhao et al., 2018 and the chicken studies by Disatham et al., 2022 differ in the approaches used to identify pathways associated with lens differentiation. Firstly, Zhao et al., 2018 sought to identify pathways enriched in lens epithelial cells compared to fiber cells across four developmental stages: E14.6, E16.5, E18.5, and P0.5. Meanwhile, Disatham et al., 2022 and Chauss et al., 2014 examined embryonic chicken lenses at a single developmental time point (E13) where the organelle free zone is actively being formed. Therefore, some of the identified differences in signaling pathways between the two studies is due to the different choices in embryonic lens developmental stages. Secondly, these studies differ in the databases and analytical methodologies used to identify enriched signaling pathways in lens epithelial cell versus fiber cell populations. These differences lead to difficulties in making a complete cross comparison between mouse and chicken lenses in these similar studies. Thus, to more accurately identify conserved signaling pathways during lens differentiation, the publicly available data from both the 2022 Disatham et al. (chicken embryonic day 13) and 2018 Zhao et al. (limited to the mouse E16.5 data) studies were reanalyzed using the same methodology described in detail in Section 5 of this review. Mouse lenses at E16.5 undergo active OFZ formation in a manner similar to that observed in chicken lenses at E13. Therefore, these developmental stages are most likely to express similar gene expression patterns and signaling pathways required for lens differentiation and formation of the OFZ.

## 3. Analysis of Chromatin Landscape in Lens Tissues with ATAC-seq

**Figure 3:**
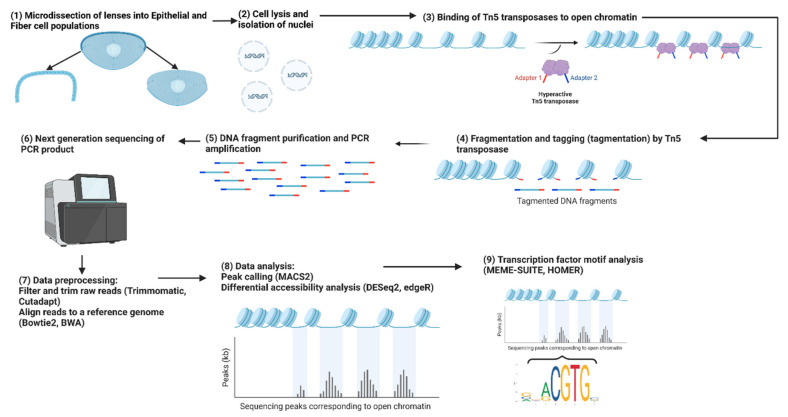
Workflow for an ATAC-seq experiment comparing lens epithelial cells and fiber cells. The figure depicts the step-by-step process of the ATAC-seq experiment, starting with the isolation of lens epithelial and fiber cells, followed by nuclei isolation, fragmentation and tagmentation, library preparation, sequencing, data preprocessing, identification of chromatin-accessible regions, and downstream transcription factor consensus sequence analysis to reveal potential regulatory elements and transcription factors involved in lens differentiation. *Adapted from “ATAC Sequencing” by BioRender.com (2023)*. Retrieved from https://app.biorender.com/biorender-templates.

Assay for transposase-accessible chromatin using sequencing (ATAC-seq) has been used to study the chromatin accessibility changes during lens differentiation [101,103,133]. It involves the use of Tn5 transposase to insert sequencing adapters into open chromatin regions, which are subsequently sequenced to determine the location of the transposase insertion sites [134,135] (Figure 3). This technique has several advantages over traditional methods of chromatin accessibility profiling, such as DNase I hypersensitivity analysis [136,137] and FAIRE-seq (formaldehyde-assisted isolation of regulatory elements with sequencing) [138,139], including its high sensitivity, low input requirement, and fast turnaround time. By profiling the open chromatin landscape of undifferentiated lens epithelial cells and terminally differentiated lens fiber cells, researchers have identified key regulatory elements and transcription factors involved in lens differentiation.

### 3.1. Changes in Chromatin Accessibility in Different Chicken and Mouse Lens Compartments 

A 2019 study by Zhao et al. used ATAC-seq to profile the chromatin accessibility of lens epithelial cells and fiber cells in mice at E14.5 and P0.5 [103]. A similar study by Disatham et al., 2019 mapped the chromatin landscape of the E13 chicken lens microdissected into the four distinct regions of lens differentiation described above that included two lens epithelial cell regions (EC, EQ) and two lens fiber cell regions (FP, FC) [101]; the key features of each region are described above. These studies were the first to establish the genome-wide chromatin accessibility changes occurring during lens differentiation. They identified gene expression patterns strongly correlated with these changes and uncovering a set of transcription factors that are predicted to bind to the accessible chromatin regions to regulate the lens differentiation process.

A cross-species comparison of chromatin accessibility changes that occur during lens differentiation of both chicken and mice is valuable for identifying evolutionarily conserved genes with proximal chromatin accessibility changes associated with lens differentiation. The identification of genes with either epithelial-cell-specific or fiber-cell-specific accessible chromatin regions in promoters of both chicken and mouse genes reveals the chromatin landscapes that are critical for driving the lens differentiation process. A unified comparison of lens differentiation-state specific open chromatin regions contained within chicken and mouse gene promoters (−7.5 kb to +2.5 kb from gene transcription start sites) was conducted. Gene promoters that contained at least one epithelial-cell-specific open chromatin region (ATAC-seq log_2_(fold change Fibers/Epi) < 0, q < 0.05) and zero fiber cell-specific open chromatin regions (ATAC-seq log_2_(fold change Fibers/Epi) > 0, q < 0.05) were classified as epithelial cell preferred gene promoters; if the reverse was true, they were classified as fiber cell preferred gene promoters. In 2019, Zhao et al. identified 1148 epithelial cell preferred gene promoters, 2674 fiber cell preferred gene promoters, and 8165 gene promoters that had both epithelial-cell- and fiber-cell-specific open chromatin regions within E14.5 mouse lenses. Similarly, Disatham et al., 2019 identified 2120 epithelial cell preferred gene promoters, 2340 fiber cell preferred gene promoters, and 7348 gene promoters that had both epithelial-cell- and fiber-cell-specific open chromatin regions within E13 chicken lenses. A cross-comparison between E14.5 mice and E13 chicken results found that 325 genes had epithelial cell preferred promoters while 757 genes had fiber cell preferred promoters in both chicken and mouse model systems (Supplementary Table S1).

A focus of the 2018 study by Zhou et al. was to identify cis-regulatory motifs within +/−2 kb from the transcriptional start sites and compare the predicted transcription factor binding with their expression levels. These data confirmed high levels of expression of the FoxE3 [61], Pax6 [46,140,141], and AP-2α [142] transcription factors in agreement with their roles established via loss-of-function studies in mice. In reference to the fiber cell differentiation, cellular elongation and formation of cell junctions between the individual hexagonal lens fibers, accumulation of crystallin proteins, gradual degradation of subcellular organelles, and formation of the syncytium [143,144,145,146,147] are the major steps. Interestingly, during the condensation of lens fiber cell nuclei, the nascent transcription of several crystallin genes is not affected until the physical disintegration of their nuclei [148]. The motif analysis pointed to nuclear effectors of FGF-MAPK kinase signaling (Etv5, Elf1, and Elk4), BMP signaling (Smad1/5/8 and Smad4), and transcription factor Hsf4 [112].

### 3.2. Chromatin Landscape of Important Genes Encoding Lens Regulatory and Structural Proteins and Identification of Transcription Factor Binding Sequences

Some notable genes with epithelial cell preferred promoters in both chicken and mouse lenses include GJA1 (connexin 43) and BMP4 (Supplementary Table S1). GJA1 is a gap junction protein essential for lens structure and transparency [149]. BMP4 is an essential mediator of lens induction both in mice and chickens [22,25,28,150,151]. Some notable genes with fiber cell preferred promoters in both chicken and mouse lenses include CRYBA4, GPX1, SOX1, and LGSN (Supplementary Table S1). Many genes displaying differentiation-state-specific promoter chromatin conformations have yet to be examined for their potential roles in lens cell differentiation, structure, or transparency (Supplementary Table S1). Pathway and gene ontology (GO) analysis of genes with differentiation-state-specific promoter chromatin configurations was conducted using the Enrichr tool [152,153,154]. Genes with epithelial cell preferred promoters in both chicken and mouse lenses were enriched in NF-kappa B signaling, HIF1α signaling, epithelial–mesenchymal transition, KRAS signaling, regulation of cell growth, insulin-like growth factor binding, and glutathione transmembrane transporter activity (Supplementary Table S2A,B). Genes with fiber cell preferred promoters in both chicken and mouse lenses were found to be enriched in GO categories mTOR signaling, Ubiquitin-mediated proteolysis, Hippo signaling, FOXO signaling, Wnt signaling, MAPK signaling, PI3K-Akt signaling, HIF-1 signaling, hypoxia, and autophagy (Supplementary Table S2C,D). All of these noted pathways have been functionally demonstrated to be essential for lens cell differentiation, structure, homeostasis, and/or transparency [1,7,155,156,157,158,159,160]. Interestingly, HIF-1 signaling was identified as an enriched signaling pathway in both epithelial cell and fiber cell genes suggesting that hypoxia-inducible transcription factor (HIF1a) plays a critical role in regulating gene expression patterns in epithelial and fiber cells during both chicken and mouse lens differentiation and could act as both an activator and a repressor of lens gene expression. Indeed, previous studies have established a requirement for hypoxia and activation of HIF1a for the formation of the organelle-free zone (OFZ) and induced expression of a variety of lens genes [84,85,143]

A well-established method for identifying DNA-binding transcription factors that regulate gene expression patterns is coupling ATAC-seq data with parallel RNA-seq data to identify which specific chromatin accessibility regions are associated with differentiation-state-specific gene expression changes. This analysis also predicts the transcription factor regulatory networks that may regulate gene expression changes associated with lens differentiation. Analysis of the DNA sequences contained within these potential cis-regulatory regions has revealed the enrichment of several DNA binding motifs [102,103]. The 2019 study by Disatham et al. of microdissected E13 chicken lenses identified HIF1a, several forkhead transcription factors (FOXO4, FOXK1, FOXP1, etc.), CTCF, and TEAD1 as predicted factors that bind differentiation-state-specific open chromatin regions associated with differentiation-state-specific gene expression changes during lens differentiation (Table 3). Similarly, the 2019 study by Zhao et al. in E14.5 and P0.5 mice identified FOXO3, several Maf family members (Mafa, Mafg, Mafk), CTCF, Pax6, Hsf4, and Sox1 as the predicted factors (Table 3). The transcription factors Pax6 [46,144,145], Hsf4 [55,56,57], Sox1 [161], and Mafg/Mafk [72,73] have established functions while others have functions that have yet to be investigated in the lens. Pax6 is expressed in the anterior pre-placodal region that gives rise to the adenohypophyseal, olfactory, and lens placodes [162]. In studies of somatic or conditionally depleted Pax6 embryos, no lens placodes were detected in E9.5 mouse embryos [46,130,141]. Thus, further studies are required to elucidate the roles of novel transcription factors or to expand the knowledge of established transcription factors known to regulate lens differentiation, structure, and transparency.

A cross-comparison analysis of ATAC-seq data of differentiation-state-specific mouse and chicken open chromatin regions was also used to identify enriched transcription factor binding motifs found near genes with conserved differentiation-state-specific expression patterns. The findings of this cross comparison are detailed in Section 5.

Overall, evolutionarily conserved chromatin accessibility patterns in lens epithelial and fiber cells reveal a network of novel and established regulatory elements, signaling pathways, and transcription factors associated with lens differentiation common to both the chicken and mouse genomes.

## 4. Multiomics Integration Analysis: Goals and Challenges

**Figure 4:**
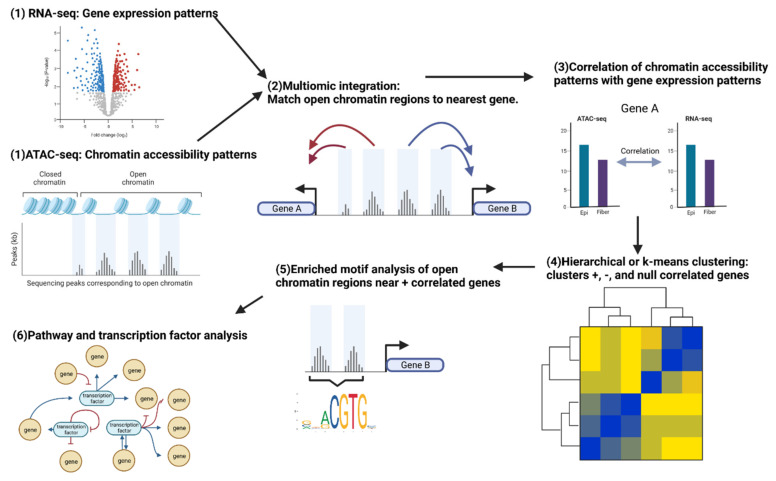
Workflow for a multiomics experiment integrating RNA-seq and ATAC-seq data comparing lens epithelial cells and fiber cells. The figure shows the integration of RNA-seq and ATAC-seq data using bioinformatics tools and statistical analysis methods, enabling the identification of potential regulatory regions, transcription factors, and gene expression patterns that contribute to lens differentiation. The integrated analysis allows for a comprehensive understanding of the molecular mechanisms underlying lens cell differentiation. *Created with BioRender.com*.

Performing multiomics integration analysis and a cross-species comparison between mouse and chicken lens differentiation models allows for a more comprehensive understanding of the regulatory mechanisms driving lens differentiation. While individual high-throughput sequencing technologies such as RNA-seq, ATAC-seq, and whole genome bisulfite sequencing (WGBS) are useful for examining particular facets of biological systems, the integration of multiple omics data can identify "real" biological regulatory mechanisms critical for differentiation, development, and disease. An example of the workflow used can be found in Figure 4.

There are several benefits to combining different types of omics data. By combining multiple types of omics data, researchers can gain a more comprehensive and detailed understanding of biological systems leading to the identification of novel regulatory mechanisms required for development, differentiation, or disease states. Another benefit of combining omics data is that it can help to overcome the limitations of individual omics data types. For example, transcriptomic data can provide information about gene expression levels, but it does not necessarily provide information about the underlying regulatory mechanisms. By contrast, epigenetic data such as ATAC-seq and WGBS can provide information about chromatin accessibility and DNA methylation, respectively, which can help to elucidate the regulatory mechanisms underlying gene expression changes.

A major challenge to integrating genomics data is that different types of omics data require different types of analysis methods, and the integration of data from multiple sources can be complex. Additionally, the different data types may not always be directly comparable, which can make data integration and analysis more difficult. Another challenge is the potential for false positives or false negatives. Combining different types of omics data can increase the risk of false positives or false negatives, as errors or biases in one data type can affect the interpretation of other data types. Therefore, it is important to carefully validate any findings from omics data integration studies using independent experimental techniques. Finally, the cost and complexity of generating and analyzing multiple types of omics data can also be a challenge. Generating multiple types of omics data can be expensive and time-consuming, and the analysis of multiple data types can be computationally intensive and require specialized expertise. Despite these challenges, integration of multiple omics datasets such as RNA-seq and ATAC-seq remains a powerful tool to identifying regulatory mechanisms, signaling pathways, and transcription factors that play a role in regulating the gene expression changes required for lens differentiation, structure, homeostasis, and transparency.

Studies by Chauss et al. in 2014 and Disatham et al. in 2022 on embryonic chicken as well as studies by Hoang et al. in 2014 and Zhao et al. in 2018 on embryonic mice used RNA-seq to measure gene expression levels and identify differentially expressed genes between lens epithelial cells and lens fiber cells. The identified evolutionarily conserved epithelial cell genes and fiber cell genes can be used to identify key regulatory mechanisms and signaling pathways associated with lens differentiation across species. Earlier studies in embryonic chicken [101] and mice [103] employed bulk ATAC-seq to map open chromatin regions and identify differentiation-state0specific chromatin accessibility changes between lens epithelial cells and fiber cells during lens differentiation. These studies examined these data using unbiased motif analyses to identify the regulatory mechanisms and signaling pathways associated with chromatin accessibility changes at prospective *cis*-regulatory regions that are directly associated with gene expression changes. These studies also identified novel candidate transcription factors that play a role in regulating gene expression changes required for lens differentiation and revealed novel regulatory mechanisms required for lens structure, homeostasis, and transparency.

In Section 5, we provide a cross-species comparison between mouse and chicken lens differentiation models to identify evolutionarily conserved mechanisms that are likely the most important for lens differentiation since they were conserved through the evolution of species. This opportunity to validate findings across different systems increases the confidence in the results, as it allows researchers to eliminate genes and mechanisms that are phenomena-specific to the species model system.

Nevertheless, there are also major challenges with combining multiomics data from different species. First, biological variability between model systems exists due to species-specific differences and due to different choices in the developmental stages of the lens. For example, we isolated E13 chicken lenses and microdissected them into four distinct cell populations (EC, EQ, FP, FC) each representing a different stage of differentiation [111]. Meanwhile, parallel mouse studies isolated and microdissected lenses into two distinct cell populations (epithelial, and fiber) [103,112]. The four developmental stages included E14.5, E16.5, E18.5, and P0.5 [112], while the other study only included P0 mice (Hoang et al., 2014). Therefore, direct comparisons of results obtained from these studies at different developmental stages and differentiation-state-specific lens regions should be interpreted with caution. A solution to this problem is to limit cross-comparison analysis to studies with the greatest similarity in developmental stage and microdissected lens populations. An example of this approach is provided in this article where RNA-seq data obtained from E13 chicken lenses microdissected into epithelial and fiber cells [102] was compared to RNA-seq data obtained from E16.5 mouse lenses microdissected into epithelial and fiber cells [112]. The reason for this is that both the E13 chicken lenses and E16.5 mouse lenses are at a developmental stage where formation of the organelle-free zone (OFZ) in the lens is still ongoing, i.e., these lens fibers have transcriptionally active nuclei (see above). Therefore, it is likely that these developmental stages in mice and chicken have a high degree of overlap in their gene expression patterns and regulatory mechanisms although this is not a perfect match. The results from this direct comparison are described in Section 5.

Another challenge to combining multiomics data from different species arises due to different analysis methods used in different studies. For example, we used the Analysis of Motif Enrichment (AME) tool [163] from MEME-Suite [164] to identify significantly enriched transcription factor binding motifs contained within open chromatin regions proximal to differentially expressed genes [101]. In contrast, mouse studies used the HOMER tool [165] to identify significantly enriched transcription factor binding motifs [103,112]. The use of alternative tools to achieve the same goal potentially introduces unintended biases in the results due to differences in the algorithms used by the respective tools. Another difference between these earlier independent studies are the differences in defining which open chromatin regions to include or exclude in subsequent analysis to identify enriched transcription factor binding motifs [101,103]. Chicken analyses averaged all open chromatin changes within 10 kb of the gene bodies and only included open chromatin regions with sums that were highly correlated with changes in gene expression to be submitted for enriched motif analysis. Meanwhile, mouse ATAC-seq data analyses separated open chromatin regions into those that align with epithelial cell gene expression patterns separated from those that align with fiber cell gene expression patterns and submitted each group separately for enriched motif analysis. Therefore, direct comparisons of ATAC-seq data obtained from these two study types should be interpreted with caution. A solution to this problem is to reanalyze the data using the same criteria for open chromatin regions and the same tool for enriched transcription factor motif analysis. An example of this is provided in this review, and the results from these comparisons are described below.

## 5. Multiomics Integration of Lens Differentiation RNA-seq and ATAC-seq: A Cross-Species Comparison

### 5.1. Evolutionarily Conserved Gene Expression Patterns Associated with Lens Differentiation

**Figure 5:**
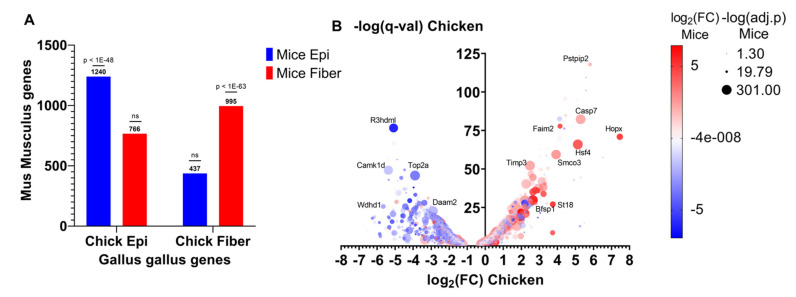
Lens differentiation-state-specific gene expression patterns are conserved between chicken and mice. (**A**) The number of *Gallus gallus* (chicken) genes classified as epithelial cell or fiber cell genes in E13 microdissected chicken lenses that were also classified as epithelial or fiber cell genes in E16.5 microdissected *Mus musculus* (mice) lenses. Epithelial cell genes are those more highly expressed in epithelial cells compared to fiber cells (log2FC < 0, q < 0.05). Fiber cell genes are those more highly expressed in fiber cells compared to epithelial cells (log2FC > 0, q < 0.05). (**B**) Volcano plot showing the genes that are differentially expressed between lens epithelial and fiber cells in E13 microdissected chicken lenses. The X-axis shows the log2FC expression level differences between fiber cells (log2FC > 0) and epithelial cells (log2FC < 0). The Y-axis shows the −log(q-val) of the significantly differentially expressed genes. Red dots indicate the genes that are also more highly expressed in lens fiber cells from E16.5 microdissected mice lenses. Blue dots indicate those that are more highly expressed in lens epithelial cells from E16.5 microdissected mice lenses. The size of the dot indicates the −log10(q-value) of significantly differentially expressed genes from microdissected mice lenses.

To identify evolutionarily conserved gene expression patterns between mice and chicken during lens differentiation, RNA-seq data obtained from E13 chicken lenses microdissected into epithelial and fiber cells [102] was compared with E16.5 mice lenses microdissected into epithelial and fiber cells [112]. A total of 2588 genes were more highly expressed in chicken lens epithelial cells (log2FC < 0, q < 0.05) compared to 1906 genes that were more highly expressed in chicken lens fiber cells (log2FC > 0, q < 0.05). In comparison, 2777 genes were more highly expressed in mice lens epithelial cells (log2FC < 0, q < 0.05) compared to 3046 genes that were more highly expressed in mice lens fiber cells (log2FC < 0, q < 0.05). A cross-species comparison revealed 1240 genes that were more highly expressed in both chicken and mice lens epithelial cells compared to 995 genes that were more highly expressed in both chicken and mice lens fiber cells (Supplementary Table S3A,B, Figure 5). A hypergeometric test was conducted to determine if there was a significant association between chicken and mice lens epithelial cell genes and also if there was a significant association between chicken and mice lens fiber cell genes. The genes classified as epithelial cell genes in chicken were significantly enriched in the list of genes classified as epithelial cell genes in mice (*p* < 1 × 10^−48^ hypergeometric test, Figure 5A). Consistently, the genes classified as fiber cell genes in chicken were significantly enriched in the list of genes classified as fiber cell genes in mice (*p* < 1 × 10^−63^, Figure 5A). Meanwhile, epithelial cell genes were not significantly associated with fiber cell genes and vice versa between mice and chicken (*p* > 0.05, Figure 5A). These results indicate that both chicken and mice lens differentiation model systems have significantly similar gene expression patterns and thus are useful for identifying evolutionarily conserved gene expression and cellular mechanisms.

Some notable evolutionarily conserved epithelial cell genes include the transcription factors Pax6 [96,131,132,141], Tfap2a [142,166], and Hes1 [16] (Figure 5); transmembrane receptors Notch1 and Notch2 [16,17] (Figure 5); gap junction transmembrane protein Gja1 (Cx43); cell surface and extracellular matrix protein fibronectin Fn1; and cyclin-dependent kinase Cdk1 (Figure 4B and Figure 5A, Supplementary Table S3A). Gja1 is a connexin expressed in the lens epithelium [153]. Cdk1 plays major role in mouse [167] and chicken [168] lens fiber cell denucleation. The 1240 evolutionarily conserved lens epithelial cell genes consist of well-established lens functional genes and those genes with potentially novel functions in the lens. 

**Figure 6:**
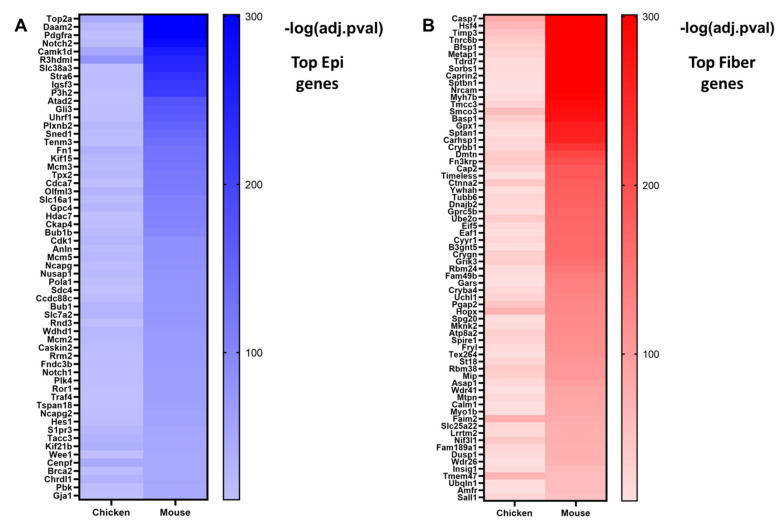
The top differentially expressed genes in mouse and chicken lens differentiation. Genes were classified as either epithelial cell genes (log2FC < 0, q < 0.05) or fiber cell genes (log2FC > 0, q < 0.05) and then ranked from lowest to highest q-value. (**A**) A heatmap showing genes in the top 200 epithelial cell genes in both E13 chicken and E16.5 mouse lenses. (**B**) A heatmap showing genes in the top 200 fiber cell genes in both E13 chicken and E16.5 mouse lenses. Darker colors represent smaller q-values.

By contrast, some notable evolutionarily conserved fiber cell genes include transcription factor Hsf4 [55,56,57]; caspase Casp7 [169]; lens-specific intermediate filament proteins Bfsp1 and Bfsp2 [124,170]; glutathione peroxidase Gpx1 [171]; multiple crystallins including Crybb1, Crygn, Cryba4, Crybb3, and Cryba1 [147]; and RNA-binding protein Tdrd7 [172] (Figure 5B and Figure 6B, Supplementary Table S3B). The 995 evolutionarily conserved lens fiber cell genes consist of both lens genes with well-established functions and those genes with potentially novel functions in the lens. Further studies would need to be conducted to elucidate their requirements for lens fiber cell function and lens differentiation.

### 5.2. Evolutionarily Conserved Cell Signaling Pathways and Gene Ontologies Associated with Lens Differentiation

**Figure 7:**
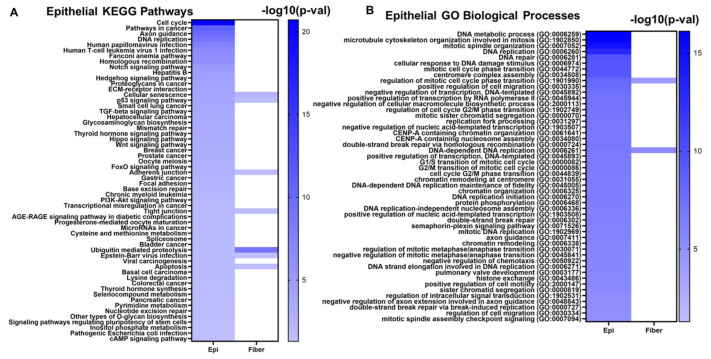
The top enriched KEGG (Kyoto Encyclopedia of Genes and Genomes) pathways (**A**) and GO Biological processes (**B**) associated with the evolutionarily conserved epithelial cell genes. Darker colors in the heatmap represent smaller *p*-values. Pathways and GO Biological processes that are also associated with fiber cell genes are also indicated in the heatmap in the “Fiber” column.

The 1240 evolutionarily conserved lens epithelial cell genes were submitted to the Enrichr tool [156,157,158] to identify significantly enriched cell signaling pathways and gene ontologies associated with lens epithelial cell function. Some notable enriched pathways included cell cycle, Notch signaling, hedgehog signaling, TGF-β signaling, Hippo signaling, Wnt signaling, FOXO signaling, and PI3K-Akt signaling (Figure 7A, Supplementary Table S4A). Similarly, some notably enriched biological processes included several cell cycle regulation processes, DNA replication, and chromatin remodeling (Figure 7B, Supplementary Table S4C). These are consistent with the cell cycle pathways identified as highly enriched pathways. These results highlight the significant roles these signaling pathways play in maintaining lens epithelial cell structure, homeostasis, and function in both mouse and chicken lenses. These processes are suppressed in epithelial cells during the lens differentiation process to form transparent mature fiber cells.

**Figure 8:**
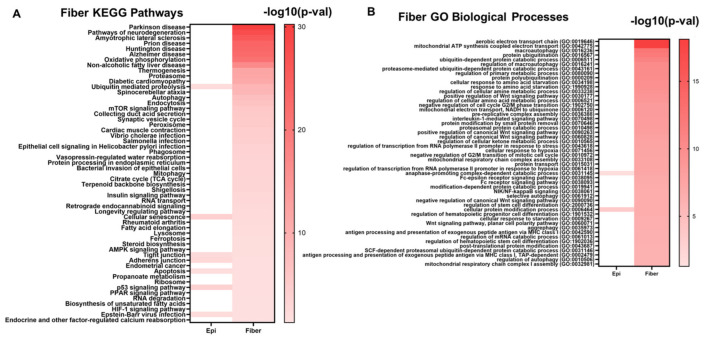
The top enriched KEGG pathways (**A**) and GO biological processes (**B**) associated with the evolutionarily conserved fiber cell genes. Darker colors in the heatmap represent smaller *p*-values. Pathways and GO Biological processes that are also associated with epithelial cell genes are also indicated in the heatmap in the “Epi” column.

The 995 evolutionarily conserved lens fiber cell genes were also submitted to the Enrichr tool to identify significantly enriched cell signaling pathways and gene ontologies associated with lens fiber cell function. Sone notably enriched pathways included pathways of neurodegeneration, oxidative phosphorylation, ubiquitin-mediated proteolysis, autophagy, mTOR signaling, mitophagy, insulin signaling, AMPK signaling, apoptosis, and HIF-1 signaling (Figure 8A, Supplementary Table S4B). Similarly, some notably enriched biological processes included macroautophagy, protein polyubiquitination, regulation of transcription in response to hypoxia, and regulation of stem cell differentiation (Figure 8B, Supplementary Table S4D). These results highlight the significant role these signaling pathways play in maintaining lens fiber cell structure, homeostasis, and function in both mice and chicken lenses. These processes are induced in fiber cells during the lens differentiation process.

### 5.3. Identification of Evolutionarily Conserved Transcription Factor Binding Motifs via Multiomics Integrated Analysis 

**Figure 9:**
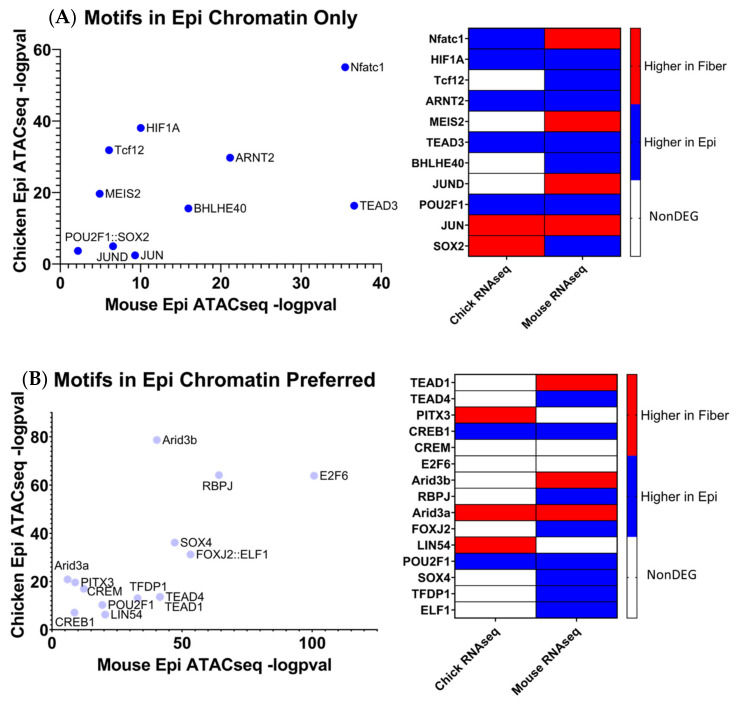
Transcription factor motifs enriched in open chromatin near evolutionarily conserved epithelial cell genes. (**A**) Transcription factor motifs enriched only in open chromatin near epithelial cell genes. (**B**) Transcription factor motifs more significantly enriched in open chromatin near epithelial cell genes as compared to fiber cell genes.

**Figure 10:**
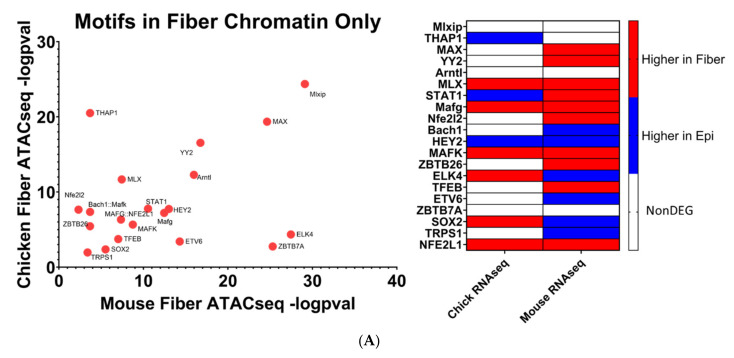

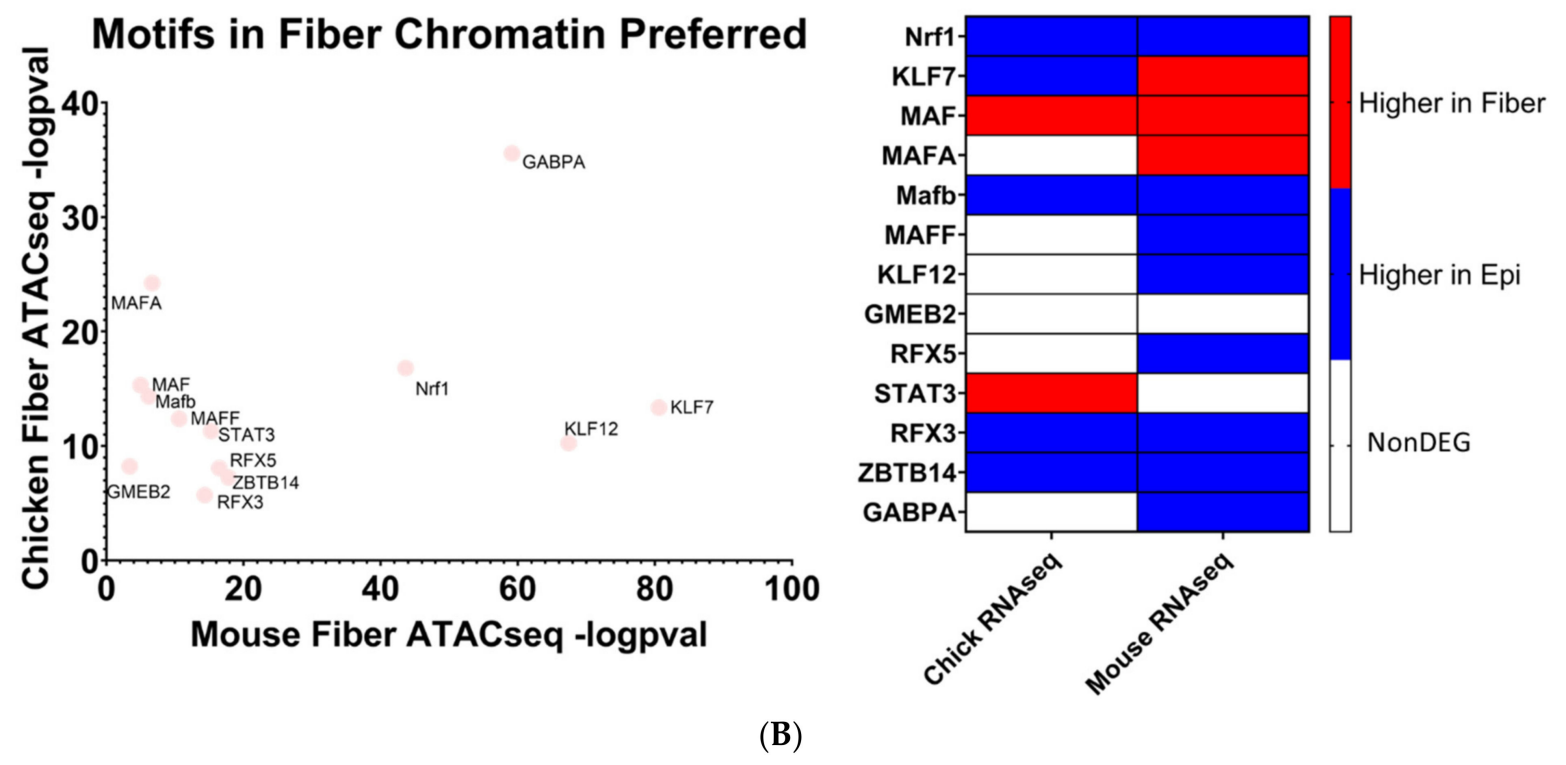
Transcription factor motifs enriched in open chromatin near evolutionarily conserved fiber cell genes. (**A**) Transcription factor motifs enriched only in open chromatin near fiber cell genes. (**B**) Transcription factor motifs more significantly enriched in open chromatin near fiber cell genes compared to epithelial cell genes.

To identify evolutionarily conserved transcription factors most associated with open chromatin regions near differentially expressed genes in lens differentiation, open chromatin regions identified in ATAC-seq data obtained from E13 microdissected chicken lenses [48] and ATAC-seq data obtained from E14.5 microdissected mouse lenses [104] were reanalyzed to identify the nearest gene transcription start site for each open chromatin region. DNA sequences contained within open chromatin regions nearest to the evolutionarily conserved epithelial cell genes and those nearest to the evolutionarily conserved fiber cell genes were separately submitted to the AME tool [163] from MEME-Suite [164] to identify the significantly enriched transcription factor motifs from the JASPAR database [173] (Figure 9 and Figure 10). Only transcription factors identified using the described analysis that were also found in both mouse and chicken lens RNA-seq are reported.

Eleven transcription factor motifs were enriched in open chromatin regions near evolutionarily conserved epithelial cell genes in chicken and mice lenses and simultaneously not enriched in open chromatin regions near evolutionarily conserved fiber cell genes, termed “Motifs in Epi only” (Figure 9A). HIF1a was identified as one of these epithelial cell specific transcription factors. Supporting evidence that HIF1a is a critical regulator of lens gene expression can be found in a 2021 study by Disatham et al. [85] that established a novel role for HIF1a-dependent gene expression in primary chicken lens epithelial cells. Although the role for TEAD3 has not yet been investigated in the lens, the data suggest that TEAD3 plays an evolutionarily conserved role in regulating lens epithelial cell gene expression patterns during lens differentiation. An additional 15 transcription factor motifs were found to be significantly more enriched in open chromatin regions near evolutionarily conserved epithelial cell genes in chicken and mouse lenses as compared to open chromatin regions near fiber cell genes, termed “Motifs in Epi preferred” (Figure 9B). RBPJ is a Notch signaling transcription factor that acts as a transcriptional activator when bound to Notch factors [16,18]. Notably, Notch1 and Notch2 are both evolutionarily conserved epithelial cell genes, suggesting that RBPJ plays an important role in the regulation of lens epithelial cell gene expression patterns [16]. A previous study established that Jag1 and RBPJ are involved in lens development [18], but the specific role for RBPJ in lens differentiation has not yet been fully elucidated.

The human genome encodes approximately 1600 DNA-binding transcription factors [174]. Given the structural similarities between their DNA-binding domains, many factors such as those with homeodomains, basic helix–loop helix, and leucine zipper DNA-binding domains recognize similar DNA motifs [175,176], and adjacent binding of two factors occurs at “suboptimal” individual sites [177]. Importantly, there is remarkable conservation of their DNA-binding specificities across 600 million years of animal evolution [178]. A nice example is the regulation of chicken δ1-crystallin gene expression by synergistic Sox2 and Pax6 binding to “suboptimal” individual sites within the 3’-located lens-specific enhancer [96]. Therefore, after individual motifs have been identified, the next step is to examine families of transcription factors and prioritizing these factors with high levels of expression in individual tissues. Our present analysis found that 20 transcription factor motifs were enriched in open chromatin regions near evolutionarily conserved fiber cell genes in chicken and mouse lenses and simultaneously not enriched in open chromatin regions near epithelial cell genes, termed “Motifs in Fiber only” (Figure 10A). An additional 13 transcription factor motifs were found to be significantly more enriched in open chromatin regions near evolutionarily conserved fiber cell genes in chicken and mouse lenses as compared to epithelial cell genes, termed “Motifs in Fiber Chromatin Preferred” (Figure 10B).

Mafg and Mafk have been established to be regulators of gene expression during embryonic lens development [72,73] where double KO mice lenses exhibit lens epithelial abnormalities and disorganized lens fibers. However, their potential roles in regulating lens fiber cell gene expression patterns during differentiation have not been fully elucidated. It is important to consider that these motifs can be also recognized by highly expressed c-Maf in lens fibers. Loss of function of c-Maf has a detrimental effect on lens differentiation and crystallin gene expression [48,68,69,70,71]. While MafA and MafB motifs have also been identified (Figure 10B), loss-of-function studies in mice did not show their requirement for normal lens development [179]. Mafg, Mafk, and their known heterodimeric factors NFE2L1 and NFE2L2 are known to regulate antioxidant response genes and autophagy [180,181]. Therefore, the unbiased identification of MAF motifs supports the methodology used to identify transcription factors regulating evolutionarily conserved fiber cell genes. 

Mlxip and MLX are heterodimer partners that are known to regulate glycolytic genes [182,183]. Their potential roles in regulating lens fiber cell differentiation have yet to be elucidated. STAT3 was previously found to be nonessential for lens development [184]. However, its specific role in regulating lens fiber cell gene expression during differentiation has yet to be fully elucidated. Likewise, identification of Bach1 motif points to another structurally similar small Maf family member Bach2 [185] which is highly expressed in the primary lens fibers of the E12.5 mouse embryos [186]. Nrf1 is another small Maf transcription factor [185] which is involved in stress regulation. Interestingly, Nrf1/Tcf11 transcriptional pathway regulates proteasome, and mutations in the PSMC3 gene encoding proteasome ATPase subunit Rpt5 are linked to early-onset cataract, as mentioned in the literature [187].

In conclusion, the use of evolutionarily conserved gene expression patterns integrated with open chromatin regions mapped proximal to these genes reveals a series of transcription factors likely regulating lens differentiation processes. Some of these transcription factors have previously established functions in lens development, but their potential roles in lens differentiation have yet to be investigated. Moreover, other predicted transcription factors have not yet been investigated in the lens and may serve as prime candidates for future experiments. This methodology of integrating RNA-seq and ATAC-seq data obtained from multiple species is a powerful tool for identifying regulatory factors required for the differentiation of the lens and can also be applied to other tissues or disease systems.

## 6. Whole-Genome Bisulfite Sequencing (WGBS) and Other Omics Opportunities to Study Conserved Regulatory Mechanisms of Gene Expression

Regulation of gene expression via DNA methylation is another mechanism to which many important DNA-binding transcription factors are sensitive, namely to cytosine methylation within the CpG dinucleotides [188,189] such as Nrf1 [190] and CTCF [191]. Importantly, our recent studies of microdissected chicken [101] and mouse [102] lenses have already demonstrated variable levels of DNA methylation within promoters and enhancers that correlate with gene expression levels in the lens fibers and lens epithelium.

**Figure 11:**
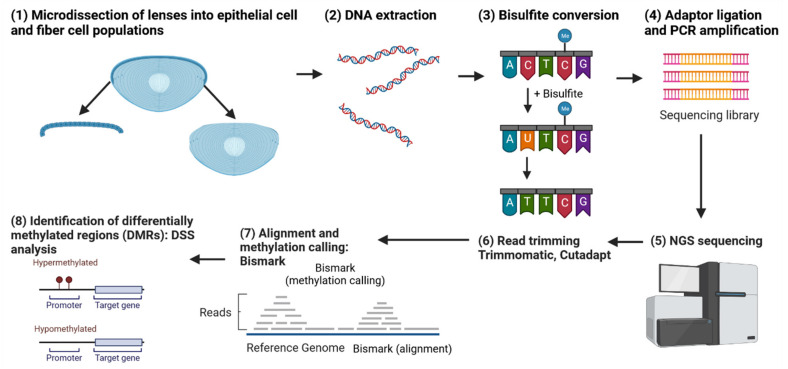
Workflow for a whole-genome bisulfite sequencing (WGBS) experiment comparing lens epithelial cells and fiber cells. The figure outlines the steps from sample preparation, bisulfite conversion, and sequencing to data preprocessing and analysis. The WGBS data are used to identify differentially methylated regions (DMRs) between lens epithelial and fiber cells, providing insights into the role of DNA methylation in lens differentiation. Additionally, the figure highlights the incorporation of transcription factor consensus sequence analysis to uncover potential regulatory elements associated with the identified DMRs, ultimately contributing to a better understanding of the epigenetic mechanisms governing lens cell differentiation. *Created with BioRender.com*.

Whole-genome bisulfite sequencing (WGBS) is a high-throughput sequencing technique used to determine the methylation status of cytosines within the entire genome (Figure 11) [192]. DNA methylation is an essential epigenetic modification that plays a crucial role in the regulation of gene expression, genomic imprinting, X-chromosome inactivation, and suppression of transposable elements [193,194,195]. By providing a comprehensive map of DNA methylation patterns, WGBS enables researchers to investigate the role of DNA methylation in various biological processes, such as development, differentiation, and disease progression.

Whole-genome bisulfite sequencing (WGBS) has been instrumental in understanding the complex mechanisms of lens differentiation, as demonstrated by two recent studies examining the role of DNA methylation in this process [102,104]. These studies have provided new insights into the relationship between DNA methylation, chromatin accessibility, and gene expression during lens differentiation.

In a study by Disatham et al. in 2022, the authors used an E13 chicken lens model to investigate the genome-wide relationships between methylation at cytosines (mCG), chromatin accessibility, and gene expression during the differentiation of eye lens epithelial cells into fiber cells. They identified over 7000 genomic loci with significant differences in mCG levels between lens epithelial and fiber cells and found that these changes in mCG levels were inversely correlated with differentiation-state-specific gene expression and chromatin accessibility [102] as quantified by RNA-seq and ATAC-seq, respectively. DNA methylation levels were measured in promoters and gene bodies of each gene in fiber cells compared to epithelial cells. Genes that had decreased levels of DNA methylation in fiber cells were associated with increased chromatin accessibility in promoters and gene bodies and increased expression levels in fiber cells. Consistently, genes that had increased levels of DNA methylation in fiber cells were associated with decreased chromatin accessibility in promoters and gene bodies and decreased expression levels in epithelial cells. Many of the genes exhibiting altered regions of DNA methylation, chromatin accessibility, and gene expression levels were associated with lens fiber cell structure, homeostasis, and transparency, including lens crystallins (CRYBA4, CRYBB1, CRYGN, CRYBB2), lens beaded filament proteins (BFSP1, BFSP2), transcription factors (HSF4, SOX2, HIF1a), and Notch signaling pathway members (NOTCH1, NOTCH2, HEY1, HES5). Moreover, the analysis of regions with cell-type-specific alterations in DNA methylation revealed an overrepresentation of consensus sequences for multiple transcription factors known to play crucial roles in lens cell differentiation, such as HIF1A [82,84,85], SOX2 [96,97,196], and the MAF family of transcription factors [68,69,70,72,97,100,197].

Similarly, another study by Chang et al., 2023 investigated the dynamics of DNA methylation and chromatin changes during mouse lens fiber and epithelium differentiation between embryos (E14.5) and newborns (P0.5) using WGBS of microdissected lenses [104]. By comparing their results with ATAC-seq and RNA-seq data, they demonstrated that reduced methylation was associated with increased expression of fiber-cell-abundant genes, including crystallins, intermediate filament proteins (Bfsp1 and Bfsp2), and gap junction proteins (Gja3 and Gja8). Furthermore, analysis of hypomethylated regions in fiber cells compared to epithelial cells revealed significantly enriched sequences that closely resembled known binding motifs for NRF1, TEAD, RXRα, Hif1α, and MafK. 

In summary, these genome-wide DNA methylation patterns between lens epithelial and fiber cells of both chicken and mouse lenses are highly associated with corresponding chromatin accessibility and gene expression patterns. Several transcription factor consensus sequences were identified in these studies. HIF1a was identified as a transcription factor bound to differentially methylated regions in both chicken and mouse lenses during lens differentiation, suggesting that HIF1a may play a conserved and significant role in the regulation of lens differentiation across species. This also implies that DNA methylation might be involved in controlling HIF1a-mediated gene expression during lens differentiation in these organisms. Indeed, previous studies have functionally validated the role of HIF1a-dependent gene regulation in lens cells [85] and have also established a requirement for the HIF1a-dependent degradation of organelles to form the lens OFZ in transparent lenses [84].

Together, these studies highlight the importance of whole-genome bisulfite sequencing in revealing the complex interplay between DNA methylation, chromatin accessibility, and gene expression during lens differentiation. The findings provide valuable insights into the molecular mechanisms underlying lens differentiation and pave the way for future investigations into the role of epigenetic mechanisms in this process.

**Figure 12:**
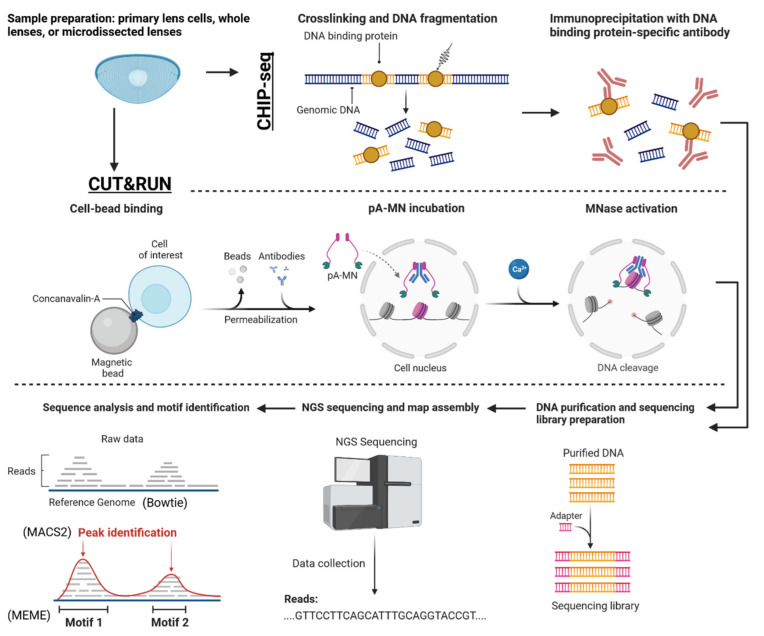
Workflow for a CUT&RUN/ChIP-seq experiment aiming to validate the genome-wide binding sites of a transcription factor previously identified as a candidate from a multiomics analysis. The figure depicts the parallel procedures for both CUT&RUN and ChIP-seq, including sample preparation, immunoprecipitation, library preparation, and sequencing. The resulting data were analyzed to identify transcription factor binding sites across the genome, providing experimental validation for the candidate transcription factor’s role in lens cell differentiation. This figure emphasizes the importance of employing complementary techniques to confirm the biological significance of findings derived from multiomics analyses. *Adapted from “CUT&RUN Procedure”, by BioRender.com (2023)*. Retrieved from https://app.biorender.com/biorender-templates.

Other recent advances in genomic technologies and their much broader feasibility allow comparative studies of gene expression across multiple species and the confirmation of transcription factor binding predicted from integration of RNA-seq and ATAC-seq data (Figure 12). As traditional ChIP-seq studies require very high numbers of cells/lens, such 140 newborn lenses to obtain the Pax6 chromatin landscape [49], and recent progress with CUT&RUN has generated data using much smaller numbers of lens cells [85]. Regarding the lens, a recent study by Disatham et al., 2021 demonstrated genome-wide binding of HIF1a in primary lens epithelial cells confirming the importance of HIF1a for lens differentiation and identifying a wealth of novel genes potentially regulated by HIF1a in the lens. 

## 7. Integrating Multiomics Analysis Results with Lens Gene Databases

RNA-seq, ATAC-seq, and other omics techniques provide a comprehensive view of the molecular landscape during lens differentiation. To identify novel mechanisms regulating lens differentiation, it is essential to integrate and compare the results from multiomics analyses with existing databases of genes with validated lens functions, such as CATMAP [198] and iSyTE [199]. CATMAP (Cataract Map) is a database of cataract-associated genes that compiles information from various sources in the literature, including human mutations and mouse models. iSyTE (integrated Systems Tool for Eye gene discovery) is a repository of lens gene expression data. One of the iSYTE resources is a list of genes highly enriched in lens tissue compared to nonlens tissue, facilitating the prioritization of lens-specific genes to identify novel regulatory mechanisms in lens development and disease.

One common method for integrating multiomics analysis results with known functional gene databases is to determine if there is a significant association between the list of differentiation-state-specific genes in lens epithelial and fiber cells compared to the list of cataract-associated genes (CATMAP) or lens-enriched genes (iSYTE). This can be evaluated using the Fisher’s Exact test of the hypergeometric test. These tests evaluate the probability that the observed overlap between the two lists occurs by chance alone. As an example, we used the list of evolutionarily conserved epithelial cell genes and fiber cell genes obtained from the cross-comparison of chicken [103] and mouse [113] lens RNA-seq to perform a hypergeometric test to determine if there was a significant association with cataract-associated genes (CATMAP) and lens-enriched genes (iSYTE) (Supplementary Table S5). The analysis revealed that 48 of 1238 (3.87%, *p* > 0.05, hypergeometric test) epithelial cell genes and 47 of 995 (4.72%, *p* < 0.01, hypergeometric test) fiber cell genes have known associations with cataracts. Additionally, 36 of 1239 (2.91%, *p* > 0.05, hypergeometric test) epithelial cell genes and 74 of 995 (7.44%, *p* < 1 × 10^−13^) fiber cell genes are known to be highly lens-enriched genes. These analyses reveal that epithelial cell genes are not as significantly associated with cataract-associated genes or lens enriched genes as compared to the significantly associated fiber cell genes. However, this does not rule out the epithelial cell gene functions required for lens differentiation, structure, and homeostasis. Indeed, several epithelial cell genes have well-defined roles in lens biology, such as Pax6. This analysis only assesses if there is an enriched relationship between the gene list of interest (epithelial or fiber cell genes) and the phenotype (cataract-associated or lens-enriched). It is also important to emphasize that evolutionarily conserved genes that are not in the CATMAP or iSYTE lens-enriched databases do not eliminate them as genes with critical functions in lens processes. Instead, the list of genes that overlap with these databases (Supplementary Table S5) should be used to provide researchers with a general guideline toward selecting candidate genes for further studies to elucidate novel functions in the regulation of lens differentiation, structure, homeostasis, and transparency.

## 8. Conclusions and Future Directions

In conclusion, omics data analysis has revolutionized the understanding of biological systems, and multiomics integration has become a powerful approach for generating a comprehensive understanding of complex biological processes. In the context of lens differentiation, multiomics integration has allowed for the identification of novel regulatory mechanisms, novel transcription factors, and epigenetic factors driving gene expression changes required for differentiation. Cross-species comparisons between mice and chicken have further elucidated evolutionarily conserved mechanisms of lens differentiation. The identification of novel gene regulatory networks (GRNs) and biological pathways provides new insights into the mechanisms that regulate lens differentiation.

Use of these technologies is in general “hypothesis-generating” research and typically leads to unexpected discoveries that are subsequently tested using gene loss-of-function and gain-of-function experiments using the most appropriate model organism(s). Another application of these methods is to compare human ES/iPS cell-derived organoids with authentic human tissues at multiple level of gene control and use of this knowledge to generate more advanced organoids for modeling of human diseases as well as for potential cell replacement therapies.

Current human genetic studies identify many variants outside of protein-coding regions or well established regulatory through mutations in 5’-UTRs of individual genes. For example, *cis*-mutations in 5′-UTR of ferritin light chain (FTL, chromosome 19q13.3) mRNA, known as the iron response element (IRE) for iron regulatory protein (IRP), which inhibits ferritin translation, cause congenital cataract [200]. Likewise, a mutation in 5’-UTR of the SLC16A12 gene causes human age-related cataract [201]. Long-range gene control via distal enhancers [202,203,204], generation of cell-specific long noncoding RNAs (lncRNAs), and generation of enhancer RNAs (eRNAs) provide mechanistic answers for the disrupted gene control and congenital defects [205,206]. 

In the last decade, remarkable progress has been made in the application of these technologies for single-cell analysis. The first breakthrough was development of drop-seq platform for single-cell transcriptomic analysis used to uncover even more complex heterogeneity of mouse retinal ganglion cells [207]. In parallel, single-cell ATAC-seq to probe chromatin landscape dynamics was established [134] and was followed by a combination of RNA-seq, ATAC-seq, and DNA methylation at the single-cell level [208]. Regarding the occupancy of chromatin analyzed by ChIP-seq and CUT&RUN, the nature of both assays requiring highly specific antibodies and other steps is unlikely to lead to single-cell data. However, precise in vivo imaging of proteins in chromatin of living or fixed individual cells at specific regions of chromatin is technically feasible with appropriate protein labels and instrumentation [209,210]. 

Not covered in this review but also important is the three-dimensional (3D) organization of individual chromosomes that can also provide clues for important gene regulatory events. Though not yet specifically established for the lens, remarkable compartmentalization patterns are involved directly or indirectly in transcriptional regulation [211]. The formation of chromatin loops can be examined by Hi-C, 4C-seq, and recent single-cell Hi-C [212,213,214]. Hi-C examines the 3D structure of DNA and chromatin by ligating DNA loci that are spatially proximate to one another and is followed by their sequencing reaching resolution between 1 and 10 kb [215]. In the lens, promoter-enhancer looping has only examined between 5′- and 3′-located distal enhancers of the mouse αA-crystallin (*Cryaa*) locus [216]. In addition, gene expression is also regulated via the interactions of RNA-binding proteins, namely with 5’-UTRs and 3’-UTRs, including their potential stem–loop–stem structures as discussed in the context of lens differentiation [147,217,218].

Despite the numerous benefits of multiomics integration analysis, there are still several limitations and potential sources of bias that need to be addressed. Technical limitations include the quality of the omics data, the choice of statistical analysis methods, and the challenges of integrating multiple types of data. However, the strengths of this approach far outweigh its limitations, and advancements in technology and analysis methods will likely continue to improve the accuracy and reliability of multiomics integration analysis.

In addition to advancing the field of lens research, the findings from multiomics integration analysis and cross-species comparisons can have broader implications for other fields. For example, the identification of novel gene regulatory networks and biological pathways can shed light on the mechanisms that drive other tissue differentiation processes. Similarly, cross-species comparisons can be used to identify evolutionarily conserved mechanisms in various biological processes, ranging from development to disease. Therefore, the integration of multiomics data and cross-species comparisons will likely continue to be a valuable tool for understanding complex biological systems and developing novel therapeutic targets in disease models.

## Figures and Tables

**Table 1:** Epithelial and fiber cell preferred genes identified with multiple RNA-seq studies in chicken and mice lenses.

Study	Model System	Notable Epithelial Preferred Genes	Notable Fiber Preferred Genes	PubmedID
Chauss et al., 2014	E13 chick lenses microdissected into 4 differentiation-state specific regions. Epithelial (EC, EQ) compared to Fiber (FP, FC)	Select Lens Crystallins (CRYAA) Cell Cycle (CDK2) Lens Signaling (FGFR2) Transcription Factors (PAX6) Mitophagy (PARL, SMURF1) Mitochondrial Regulation (BID, PPARGC1A) Macroautophagy (MAP1LC3C, PIK3CB) Mitochondrial Repair/Protection (TXNRD1)	Select Lens Crystallins (ASL1, CRYAB) Actin-Capping (TMOD1) Beaded Filaments (BFSP1, BFSP2) Lens Signaling (EPHA2, CCDC80, FZD3) Transcription Factors (HSF4, SOX2, PROX1) Cataract-associated genes (LGSN) Mitophagy (BNIP3L, BNIP3) Mitochondrial Regulation (DNAJA3, DNAJC15, SLC25A22, TFB1M, SNPH) Macroautophagy (BECN1, FYCO1, MTOR, ATG3, ATG4B, SQSTM1, WIP11) Mitochondrial Repair/Protection (GLRX, MSRA, NXNL1)	24928582
Zhao et al., 2018	E14.5, E16.5, E18.5, and P0.5 mouse lenses microdissected into Epithelial compared to Fiber	Transcription Factors (Pax6, Foxe3, Dmrta2, Mafb, Tgap2a, Tulp3) Sumoylation (Sumo1, Sumo3, Sae1, Uba2)	Crystallin Protein Translation Initiation (Eif3h, Eif3k) Transcription Factors (Stat5a, Prox1, Sox1, Maf, Hsf4, Mafg, Arid3b, Bach2, Carhsp1) Macroautophagy (Mtor, Plekhm1) Mitophagy (Bnip3)	29883638
Disatham et al., 2022	E13 chick lenses microdissected into Epithelial compared to Fiber	Crystallins (CRYBB2) Cell Cycle (CCND1) DNA Methylases (DNMT3B) DNA demethylases (TET1) Signaling (NOTCH2) Transporters (SLC2A1) Metabolism (LDHA, ENO1) Transcription Factors (HIF1a, RBPJ, HES1, MYCN, HEY1, CREB3L1, CREB3L2, FOXP1, RREB1)	Crystallins (CRYBB3, CRYBA1, ASL1, CRYGN, CRYBB1, CRYBA4) Transcription Factors (HSF4) Beaded Filaments (BFSP1, BFSP2) Mitophagy (BNIP3L) Connexins (GJA3) mRNA processing (TDRD7) Transcription Factors (SOX2, STAT3, MLX, Arid5a)	35246225
Hoang et al., 2014	P0 mouse lenses microdissected into Epithelial compared to Fiber	Tyrosine Kinase Receptors (Drd1, Pdgfra, Ror1, Ephb2, Ephb4, Erbb2, Ptk7, Tek, Pdgfrb, Axl, Ephb6, Epha7, Epha10, Kdr, Flt4, Egfr, Tie1, Ror2, Epha3, Epha1, Flt1) Notch Signaling (Notch1, Notch2, Notch3, Notch4, Jag2, Dll4, Dll1, Hes6, Hes1, Rbpj, Hes5, Numb) Wnt Signaling (Fzd1, Fzd7, Fzd2, Fzd4, Fzd8, Wnt5a, Dvl2) TGFB superfamily (Tgfbr1, Tgfbr2, Ncam1, Acvr1, Eng, Bmp1, Tgfb2, Bmp7, Inha) Dna degradation and repair (H2afx, Rad52, Lig1, Rad50, Nbn, Mdc1, Atm, Chek1, Rad51) Proteolysis (Casp2, Casp9, Xiap, Birc5) Aquaporins (Aqp1, Aqp4, Aqp8) Gap junctions (Gja1, Gjc1, Gja4) Intermediate filaments (Lmnb1, Lmnb2, Nes)	Tyrosine Kinase Receptors (Fgfr3, Epha2, Met, Ret) Notch Signaling (Jag1, Herpud1) Wnt Signaling (Wnt7a, Wnt7b, Wnt5b) TGFB superfamily (Bmpr1b) DNA degradation and repair (Dnase2b, Lig4) Proteolysis (Casp7, Birc7, Birc2) Aquaporins (Mip) Gap junctions (Gja3, Gja8, Gje1) Intermediate filaments (Bfsp1, Bfsp2)	25489224

**Table 2:** Cell signaling pathways associated with epithelial or fiber cell genes from multiple RNA-seq studies in chicken and mouse lenses.

Study	Model system	Notable Epithelial Preferred Signaling Pathways and Ontologies	Notable Fiber Preferred Signaling Pathways and Ontologies	PubmedID
Zhao et al., 2018	E14.5, E16.5, E18.5, and P0.5 mouse lenses microdissected into Epithelial compared to Fiber	Cell cycle, cell adhesion, signal transduction, DNA repair, DNA methylation, extracellular matrix organization, cell differentiation, cell migration, Wnt signaling, Sumoylation Pathway, Notch signaling, TGFB signaling, BMP signaling, RAR activation, ATM signaling, Human embryonic stem cell pluripotency	Translation initiation, vesicle-mediated transport, oxidation-reduction process, mitochondrial translation, lipid metabolism, autophagy, protein ubiquitination, mitochondrial dysfunction, EIF2 signaling, Oxidative phosphorylation, mTOR signaling, PI3K/Akt signaling, Glycolysis	29883638
Disatham et al., 2022	E13 chick lenses microdissected into Epithelial compared to Fiber	Extracellular matrix organization, collagen fibril organization, eye development, epithelial mesenchymal transition, unfolded protein response, G2-M checkpoint, cell cycle control, Notch signaling	Oxidative phosphorylation, Apoptosis, mTORC1 signaling, cholesterol homeostasis, TGF-beta signaling, hypoxia, heme metabolism, intermediate filament organization, hedgehog signaling	35246225
Hoang et al., 2014	P0 mouse lenses microdissected into Epithelial compared to Fiber	Cell cycle, cell division, cell migration	Lens development in camera-type eye	25489224

**Table 3:** Transcription factors with enriched binding motifs in open chromatin regions mapped with ATAC-seq studies in mice and chicken lenses.

Study	Model System	Transcription Factors Associated with Differentially Expressed Genes	PubmedID
Disatham et al., 2019	E13 chick lenses microdissected into 4 differentiation-state specific regions. Epithelial (EC, EQ) compared to Fiber (FP, FC)	NFATC2, IRF1, NFAT5, ZNF384, FOXP2, CTCF, FOXK1, FOXP1, FOXK2, Arid3b, NFATC3, RBPJ, E2F6, SP2, NFATC1, ARNT::HIF1a, Hoxd9, FOXO4, Foxj2, MEF2C, Myod1, TEAD1, SPIC, FOXI1, FOXB1	31136738
Zhao et al., 2019	E14.5 and P0.5 mice lenses microdissected into Epithelial compared to Fiber	Etv5, Foxk2, Foxn3, Foxo3, Gata3, Gatad1, Hsf4, Jun, Maf, Mafa, Mafg, Mafk, Meis2, Mycl, Mycn, Nf2, Nfat5, Rxra, Smad1, Smad4, Sox1, Sox13, Tead1, Prox1, Foxj3, Yy1, Nfatc1, Sox8, Sp1, CTCF, Pax6	31053165

## Supplementary Materials

The following are available online at https://www.mdpi.com/article/10.3390/biom13040693/s1, Table S1. Epithelial and fiber cell gene promoters in chicken and mice. Lists of gene symbols for genes with epithelial-cell-specific or fiber-cell-specific promoters (−7.5 kb to +2.5 kb from gene transcription start sites) in both chicken and mouse lenses. Supplementary Table S2. Enrichr analysis of conserved Epi and Fiber gene promoters. A. KEGG pathways associated with Epi gene promoters. B. GO biological processes associated with Epi gene promoters. C. KEGG pathways associated with fiber gene promoters. D. GO biological processes associated with fiber gene promoters. Supplementary Table S3. Conserved Epi and fiber genes. A. Genes more highly expressed in epithelial cells compared to fiber cells in both chicken and mice. Ranked by lowest to highest q-value. B. Genes more highly expressed in fiber cells compared to epithelial cells in both chicken and mice. Ranked by lowest to highest q-value. Supplementary Table S4. Enrichr analysis of conserved Epi and fiber genes. KEGG pathways associated with genes more highly expressed in epithelial cells (A) or fiber cells (B) in both chicken and mouse lenses. GO biological processes associated with genes more highly expressed in epithelial cells (C) or fiber cells (D) in both chicken and mouse lenses. Supplementary Table S5. CATMAP and iSYTE overlap of conserved Epi and fiber genes. A. Genes more highly expressed in epithelial cells in both chicken and mice that are also in the CATMAP or iSYTE highly enriched lens database. B. Genes more highly expressed in fiber cells in both chicken and mice that are also in the CATMAP or iSYTE highly enriched lens database.

## Abbreviations

**Abbreviation**	**Definition**
ATAC-seq	Assay for transposase accessible chromatin with sequencing
WGBS	Whole-genome bisulfite sequencing
CHIP-seq	Chromatin immunoprecipitation with sequencing
CUT&RUN	Cleavage under targets and release using nuclease
EST	Expressed sequence tags
EC	Anterior central epithelial cells
EQ	Equatorial epithelial cells
FP	Cortical fiber cells
FC	Central fiber cells
FAIRE-seq	Formaldehyde-assisted isolation of regulatory elements
GO	Gene Ontology
Epi	Lens epithelial cells
Fiber	Lens fiber cells
KEGG	Kyoto Encyclopedia of Genes and Genomes
AME	Analysis of motif enrichment
